# Genomic prediction using preselected DNA variants from a GWAS with whole-genome sequence data in Holstein–Friesian cattle

**DOI:** 10.1186/s12711-016-0274-1

**Published:** 2016-12-01

**Authors:** Roel F. Veerkamp, Aniek C. Bouwman, Chris Schrooten, Mario P. L. Calus

**Affiliations:** 1 Animal Breeding and Genomics Centre, Wageningen UR Livestock Research, P.O. Box 338, 6700 AH Wageningen, The Netherlands; 2 Department of Animal and Aquacultural Sciences, Norwegian University of Life Sciences, P.O. Box 5003, 1432 Ås, Norway; 3 CRV BV, P.O. Box 454, 6800 AL Arnhem, The Netherlands

## Abstract

**Background:**

Whole-genome sequence data is expected to capture genetic variation more completely than common genotyping panels. Our objective was to compare the proportion of variance explained and the accuracy of genomic prediction by using imputed sequence data or preselected SNPs from a genome-wide association study (GWAS) with imputed whole-genome sequence data.

**Methods:**

Phenotypes were available for 5503 Holstein–Friesian bulls. Genotypes were imputed up to whole-genome sequence (13,789,029 segregating DNA variants) by using run 4 of the 1000 bull genomes project. The program GCTA was used to perform GWAS for protein yield (PY), somatic cell score (SCS) and interval from first to last insemination (IFL). From the GWAS, subsets of variants were selected and genomic relationship matrices (GRM) were used to estimate the variance explained in 2087 validation animals and to evaluate the genomic prediction ability. Finally, two GRM were fitted together in several models to evaluate the effect of selected variants that were in competition with all the other variants.

**Results:**

The GRM based on full sequence data explained only marginally more genetic variation than that based on common SNP panels: for PY, SCS and IFL, genomic heritability improved from 0.81 to 0.83, 0.83 to 0.87 and 0.69 to 0.72, respectively. Sequence data also helped to identify more variants linked to quantitative trait loci and resulted in clearer GWAS peaks across the genome. The proportion of total variance explained by the selected variants combined in a GRM was considerably smaller than that explained by all variants (less than 0.31 for all traits). When selected variants were used, accuracy of genomic predictions decreased and bias increased.

**Conclusions:**

Although 35 to 42 variants were detected that together explained 13 to 19% of the total variance (18 to 23% of the genetic variance) when fitted alone, there was no advantage in using dense sequence information for genomic prediction in the Holstein data used in our study. Detection and selection of variants within a single breed are difficult due to long-range linkage disequilibrium. Stringent selection of variants resulted in more biased genomic predictions, although this might be due to the training population being the same dataset from which the selected variants were identified.

**Electronic supplementary material:**

The online version of this article (doi:10.1186/s12711-016-0274-1) contains supplementary material, which is available to authorized users.

## Background

Genomic selection is increasingly applied in breeding programs for livestock species, e.g. [1, 2], and has led to dramatic increases in genetic progress [3], especially in dairy cattle. However until now, accuracies of genomic prediction are still not close to 1, although one of the expectations was that, compared to the currently used common single nucleotide polymorphism (SNP) panels, whole-genome sequence data would increase accuracies of genomic prediction. Because most of the causal mutations that underlie quantitative trait loci (QTL) are expected to be included as genetic markers in the sequence data, it is expected that causal mutations will be identified more precisely than with the common lower density SNP chips [4] and that the reliability of genomic predictions and its persistency across generations and even across breeds [5, 6] will improve. This was confirmed on simulated data [7], but in practice, the use of cattle and chicken sequence data has not increased the reliability of genomic predictions [8, 9].

Several reasons may explain why the accuracy of genomic predictions does not increase when sequence data is used: (1) if the number of training individuals is small, the effects of QTL may be estimated with too large errors and thus, little advantage is gained by using sequence data [10]; (2) if training is performed within a breed or line, long-range linkage disequilibrium (LD) may prevent the precise localisation of quantitative trait nucleotides (QTN) when all sequence variants are fitted simultaneously [8]; and (3) many different linear combinations of variants (that are in high LD) may occur and result in equally accurate genomic predictions for the same set of phenotypes. Therefore, it is not possible to construct a unique prediction equation and no benefit can be expected from using more precise measures at the DNA level (i.e. more variants). In fact, it might be better to use fewer variants that are located closer to the QTN, than to rely on the complex LD structure between variants for the prediction of selection candidates. This was also found in a simulation study for across-breed prediction by Wientjes et al. [11].

These previous studies raise several questions i.e. how much of the total genetic variation is tagged by using different sets of variants, from commercial SNP chips up to whole-genome sequence data and to variants selected from a genome-wide association study (GWAS) using (imputed) sequence data, and how is accuracy of genomic prediction affected. Our objective was to compare the proportion of variance explained and the accuracy of genomic prediction based on imputed sequence data, lower density SNP panels, and preselected variants from a GWAS based on imputed whole-genome sequence.

## Methods

### Phenotypes

De-regressed proofs (DRP) were available for somatic cell score (SCS), interval between first and last insemination (IFL), and protein yield (PY) for 5503 Holstein–Friesian bulls provided by CRV (Arnhem, the Netherlands). DRP were calculated according to [12]:

$${\text{DRP}} = {\text{PA}} + \left( {{\text{EBV}} - {\text{PA}}} \right) *\left( {\frac{{{\text{EDC}}_{\text{EBV}} }}{{{\text{EDC}}_{\text{prog}} }}} \right),$$

where ${\text{EBV}}$ is the estimated breeding value of a bull for a trait available from the national evaluations, and ${\text{PA}}$ is the parent average of the bull for that trait. Effective daughter contribution, ${\text{EDC}}_{\text{EBV}}$, represents the effective number of daughters with phenotypes that contributed to the ${\text{EBV}}$ of a bull [13] and was calculated according to [12] as $\alpha *{\text{REL}}_{\text{EBV}} /(1 - {\text{REL}}_{\text{EBV}} )$, where ${\text{REL}}_{\text{EBV}}$ is the published reliability for ${\text{EBV}}$ and $\alpha = (4 - h^{2} )/h^{2}$, where $h^{2}$ is the heritability of the trait. ${\text{EDC}}_{\text{prog}} = {\text{EDC}}_{\text{EBV}} - {\text{EDC}}_{\text{PA}}$, where ${\text{EDC}}_{\text{PA}} = \alpha {\text{REL}}_{\text{PA}} /(1 - {\text{REL}}_{\text{PA}} )$ and ${\text{REL}}_{\text{PA}} = ({\text{REL}}_{\text{sire}} + {\text{REL}}_{\text{dam}} )/4$ [14]. As the number of daughters with phenotypic information for a trait increases, the reliability of the ${\text{EBV}}$ of a bull and ${\text{EDC}}_{\text{EBV}}$ increase. The average ${\text{EDC}}_{\text{EBV}}$ (and its range) for animals in the training set was equal to 266 (24 to 971) for SCS, 643 (47 to 4851) for IFL, and 245 (24 to 693) for PY.

Following van Binsbergen et al. [8], the bulls were assigned either to the population used for variant detection (discovery population) in the GWAS (and training for genomic prediction) or to the validation population. Assignment was based on year of birth, bulls born before 2001 (3416 bulls) were assigned to the discovery population and bulls born between 2001 and 2008 (2087 bulls) to the validation population.

### Genotypes

In total, 551 of the bulls in this study were genotyped with the Illumina BovineHD BeadChip (Illumina Inc., San Diego) and the other 4952 bulls were genotyped with a 50k SNP panel and imputed to BovineHD (734,403 SNPs). Imputation from the 50k panel to the BovineHD SNP panel was performed with BEAGLE 3.3.0 [15, 16], using additional Holstein bulls in a reference set of 1333 animals genotyped with the BovineHD SNP panel. For this first step, the error rate of imputation was low (with a slightly smaller reference population, it was equal to 0.41%) [17]. The HD genotypes of the bulls were imputed to whole-genome sequence using the sequenced population from the 1000 Bull Genomes Project Run 4 as reference population. This multi-breed reference population included 1147 sequenced animals with on average an 11-fold coverage, among which 311 Holstein bulls. All the individuals were used as reference because earlier studies showed that a multi-breed sequenced reference population can be beneficial for imputation accuracy, especially for SNPs with a low minor allele frequency (MAF) [4, 18, 19]. Polymorphic sites, including SNPs and short insertions and deletions (InDels), were identified for the 1147 individuals simultaneously using the multi-sample approach implemented in SAMtools’ mpileup along with BCFtools as described in Daetwyler et al. [4]. Genotype calls for the 1000 Bull Genomes reference population were improved with BEAGLE [15] using genotype likelihoods from SAMtools and inferred haplotypes in the samples. The sequence data contained 36,916,855 bi-allelic variants of which 30,339,468 had four or more copies of the minor allele in the reference population and were used for imputation. Imputation of HD genotypes to whole-genome sequence was done using standard settings in MINIMAC2 [20] and the pre-phased reference genotypes that resulted from BEAGLE. MINIMAC2 gave empirical imputation reliabilities (squared correlations between imputed and true genotypes for typed SNPs only) of 0.99 on average per chromosome including only the SNPs with MAF higher than 0.01, except for chromosomes 2, 4, 5, 26, and 27 where the average per chromosome ranged between 0.63 and 0.66.

### GWAS using sequence information

For the three traits analyzed, the bulls in the discovery set were used to perform a GWAS in which each variant was fitted separately and a genomic relationship matrix (GRM) based on the BovineHD SNPs was constructed to account for population structure. The mixed linear model based association analysis (MLMA) in the package GCTA [21] was used. All sequence variants (SNPs and biallelic InDels) with a MAF higher than 0.01 (n = 13,789,029) were tested for their association. The model was:

$${\mathbf{y}} = 1\mu + {\mathbf{Zg}} + {\text{b}}{\mathbf{x}} + {\mathbf{e}},$$

where ${\mathbf{y}}$ is the vector of DRP of all individuals, $\mu$ is the overall mean, **1** is a vector of ones, ${\mathbf{Z}}$ is an incidence matrix that links records to bulls, ${\mathbf{g}}$ is a vector of the genomic breeding values of all individuals, ${\text{b}}$ is the additive (fixed) effect of the candidate variants to be tested for association, ${\mathbf{x}}$ is a vector of the variants’ genotype indicator variable coded as 0, 1 or 2, and ${\mathbf{e}}$ is a vector of random residuals. Genomic breeding values were assumed to be distributed as ${\mathbf{g}}|{\mathbf{GRM}},\sigma_{g}^{2} \sim N({\mathbf{0}}, {\mathbf{GRM}}\sigma_{g}^{2} )$, where **GRM** is the genomic relationship matrix calculated from the variants present on the BovineHD chip, and $\sigma_{g}^{2}$ is the additive genetic variance picked up by the markers. For ease of computation, $\sigma_{g}^{2}$ is estimated based on the null model without variants and then fixed while testing for the association between each of the variants and the trait. Diagonal and off-diagonal values of the **GRM** were calculated following [22, 23] as:

$$\begin{aligned} GRM_{jk} &= \frac{1}{N}\mathop \sum \limits_{i} GRM_{ijk} \\ &= \left\{ {\begin{array}{l} {\frac{1}{N}\mathop \sum \limits_{i} \frac{{\left( {x_{ij} - 2p_{i} } \right)\left( {x_{ik} - 2p_{i} } \right)}}{{2p_{i} \left( {1 - p_{i} } \right)}},\quad j \ne k} \\ {1 + \frac{1}{N}\mathop \sum \limits_{i} \frac{{x_{ij}^{2} - \left( {1 + 2p_{i} } \right)x_{ij} + 2p_{i}^{2} }}{{2p_{i} \left( {1 - p_{i} } \right)}},\quad j = k} \\ \end{array} } \right. \\ \end{aligned}$$

where $GRM_{ijk}$ is the estimated relationship between individuals $j$ and $k$ at locus $i$, and $N$ is the number of variants. The genotypes of the variants ($x_{i}$) were coded as 0, 1 or 2, and $p_{i}$ is the allele frequency of the allele for which the homozygous genotype was coded as 2. Residual effects were assumed to be distributed as ${\mathbf{e}}|\sigma_{e}^{2} \sim N({\mathbf{0}},{\mathbf{I}}\sigma_{e}^{2} )$, where $\sigma_{e}^{2}$ is the residual variance. This is not the usual estimate of the residual variance associated with an individual phenotype, but the residual of the DRP. Ideally the DRP should have been weighted according to the ${\text{EDC}}$ but this was not feasible in GCTA.

Manhattan plots were created using the R package qqman [24] excluding all variants with a −log_10_(p) less than 1 for computational ease.

### Selection of variants

From the full set of 13,789,029 imputed sequence variants (ISQ), SNPs that were present on the two commonly used SNP panels (50k or Bovine HD) were selected as subsets. Then, the GWAS results for the discovery population were used to select 10 subsets of variants within ISQ, HD and 50k variants, which totalled 33 sets of variants. Selection was “*p* value based” using arbitrary cut-off levels of 3 and 5 for the −log_10_(p). A disadvantage is that selecting variants purely on a −log10(p) threshold results in many variants in one region being selected for genomic prediction, due to LD between variants and to running regression on each variant separately. Therefore, we also carried out a conditional and joint GWAS (COJO) using the results from the single-variant GWAS model [25]. In the COJO analysis, variants were added to the model one by one, starting with the variants that had the most significant effect, on the basis of joint and conditional significance level from the GWAS. The joint and conditional significant level is the significance level from the GWAS results conditional on the LD with the selected variants already in the model and the joint significance level of these variants. Variants were added to the model only when more variance was explained compared to that obtained with the other variants already in the model. By performing the COJO, the large number of variants led to computing limitations for the calculation of LD and selection from all variants, while the large family structures of the dataset led to over-fitting due to high collinearity between the variants. Collinearity resulted in grossly overestimated conditional variant effects and inflated p values. In order to circumvent these two issues, four different COJO analyses were performed. For the first two analyses, all variants with a −log_10_(p) less than 3 were a priori removed and variants that were in LD (defined as r^2^) with the variants already in the model with an r^2^ higher than 0.8 were not added in the forward selection step. Then, variants were selected based on a conditional and joint significance level −log_10_(p) greater than 3 (COJO3) or 5 (COJO5). For the third and fourth COJO analyses, all variants were considered a priori, but it was assumed that variants that were more than 100 Mb apart were in complete linkage equilibrium. Thus, estimated effects were not adjusted for loci that were more than 100 Mb apart since they are assumed to be independent, and the LD threshold was only applied for loci that were less than 100 Mb apart. Also, variants that were in LD with the variants already in the model with an r^2^ higher than 0.5 were not added. Then, variants were selected based on a conditional and joint significance level −log_10_(p) higher than 5 (COJO5LD) or the 100 variants with the largest effect were selected (COJO#100).

### Variance explained by the selected variants

The first method to evaluate the value of the selected variants consisted in estimating the variance of the DRP that can be explained by the variants for the validation animals. The estimated genetic variance was expressed as a proportion of the total variance i.e. the so-called genomic heritability (h^2^) [26] as relevant for the DRP. This heritability is not directly comparable to the usual heritability of a single phenotypic record since the estimated residual variance is not directly comparable with the residual variance of a single phenotypic record. Using the 33 sets of variants (Tables 1 and 2), the 33 ${\mathbf{GRM}}$ were calculated following [22] as described above, and variance components were estimated using GREML in GCTA. Variances were not only estimated using the ${\mathbf{GRM}}$ for the subsets of selected variants, but also by using a complementary ${\text{GRM}}$ (${\mathbf{GRMc}}$) based on the remaining variants that were not selected for inclusion in the ${\mathbf{GRM}}$ for the subsets of selected variants. For example, the 50k panel contained 49,580 variants, thus the remaining 13,739,449 (=13,789,029 − 49,580) variants were used to create the complementary ${\mathbf{GRMc}}$. For the scenarios with variant selection based on the 50k SNP or Bovine HD panels only, no additional pruning on LD was performed for the **GRMc**. For the “p value based variant selection” (Table 1), a GREML analysis with these ${\mathbf{GRMc}}$ matrices reflected the variance explained by variants without an association with the trait. For the subsets selected with the COJO analysis (Table 2), many variants in high LD with the selected variants were still present in the remaining set. Therefore, an additional step was performed to exclude from the ${\mathbf{GRMc}}$ not only the selected variants but also the variants that were in significant LD (p < 0.01) with the selected variants. GCTA was used to search for variants in significant LD with the selected variants [21], and the search was limited to a 2-Mb window on either side of each selected variant.

**Table 1:** Number of variants in each of the subsets of variants selected from the SNP panels and selection criteria

Trait	Selection criteria	Imputed sequence (ISQ)	HD	50k
	All variants	13,789,029	656,044	49,580
PY	−log10(p) > 3	24,387	1238	120
	−log10(p) > 5	2,194	159	27
SCS	−log10(p) > 3	23,346	1203	98
	−log10(p) > 5	1539	90	7
IFL	−log10(p) > 3	22,833	987	61
	−log10(p) > 5	853	27	4

**Table 2:** Number of variants in each of the subsets of variants selected using COJO, and number of variants in linkage disequilibrium (LD) with the selected variants, which were ignored in the GRMc

Trait	Selection criteria	Number of variants in subset of selected variants	Number of variants in LD with selected variants
PY	COJO3	90	1,650,152
	COJO5	64	1,154,416
	COJO5LD	35	615,586
	COJO#100	100	1,688,270
SCS	COJO3	195	3,241,932
	COJO5	215	3,449,212
	COJO5LD	42	757,095
	COJO#100	100	1,652,678
IFL	COJO3	264	3,835,730
	COJO5	209	3,151,538
	COJO5LD	35	607,631
	COJO#100	100	1,675,727

Finally, each ${\mathbf{GRM}}$ was fitted together with its ${\mathbf{GRMc}}$ to obtain a more conservative and probably better estimate of the variance explained by the selected set of variants. When fitting multiple ${\mathbf{GRM}}$, GREML will partition the variances according to the maximum likelihood.

### Accuracy of genomic predictions with selected variants

The second method to evaluate the value of the selected variants consisted in calculating genomic predictions for the validation animals (GEBV) and correlating these with the phenotypes of the same animals. Since GCTA excluded by default the animals without phenotypes from the ${\mathbf{GRM}}$, several steps were required. First, using GREML in GCTA, each of the 33 ${\mathbf{GRM}}$ was fitted separately for the discovery (training) animals. Secondly, BLUP solutions for the effects of variants were back-solved from the GEBV for the discovery animals. Finally, using the package PLINK (v1.90b3c 64-bit; 2 Feb 2015; http://pngu.mgh.harvard.edu/purcell/plink/; [27]), the BLUP solutions for the effects of variants were used to calculate the GEBV for the validation animals. Similar to the estimation of the variance, GEBV for the validation animals were also computed using both the ${\mathbf{GRM}}$ and the complementary ${\mathbf{GRMc}}$ simultaneously in the GREML model, followed by back-solving the effects of variants and calculating the breeding values with the combined solutions.

Accuracy of genomic prediction was calculated for the validation animals as the correlation between the DRP and GEBV for the different traits, assuming that the DRP was based on very many progeny. Furthermore, the regression coefficient of the DRP on the GEBV was calculated to evaluate the bias of predictions.

## Results

### GWAS

**Fig. 1:**
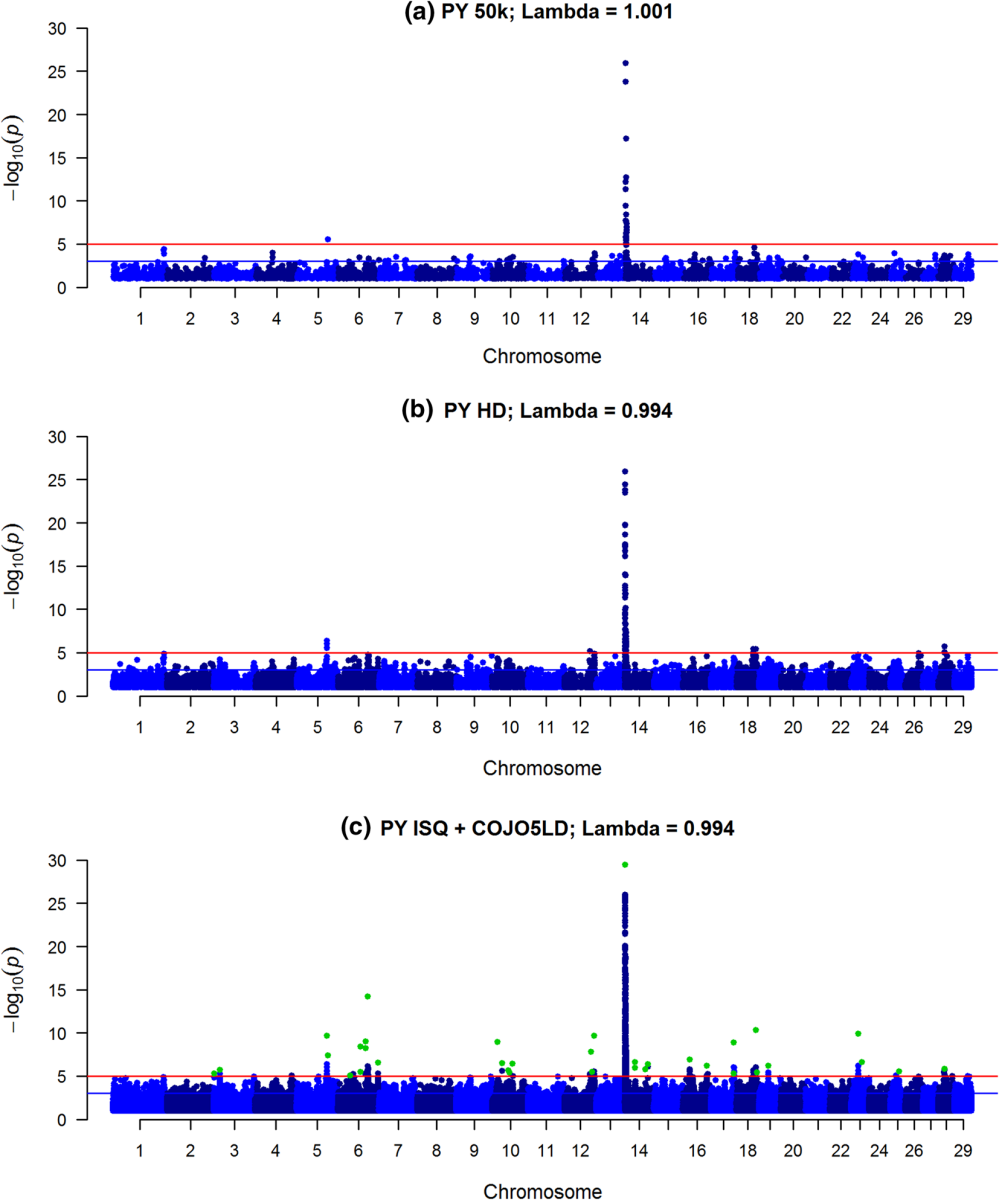
Manhattan plot for protein yield (PY) using Bovine 50k (**a**), BovineHD (**b**) and ISQ data and variants after selection (COJO5LD in *green*) (**c**). Significance of variants effects (−log10(p)) based on the GCTA single variant analyses for protein yield (PY) using Bovine 50k (**a**), BovineHD (**b**), and full sequence data (ISQ) and the variants selected after the COJO5LD analysis (*green*) (**c**)

**Fig. 2:**
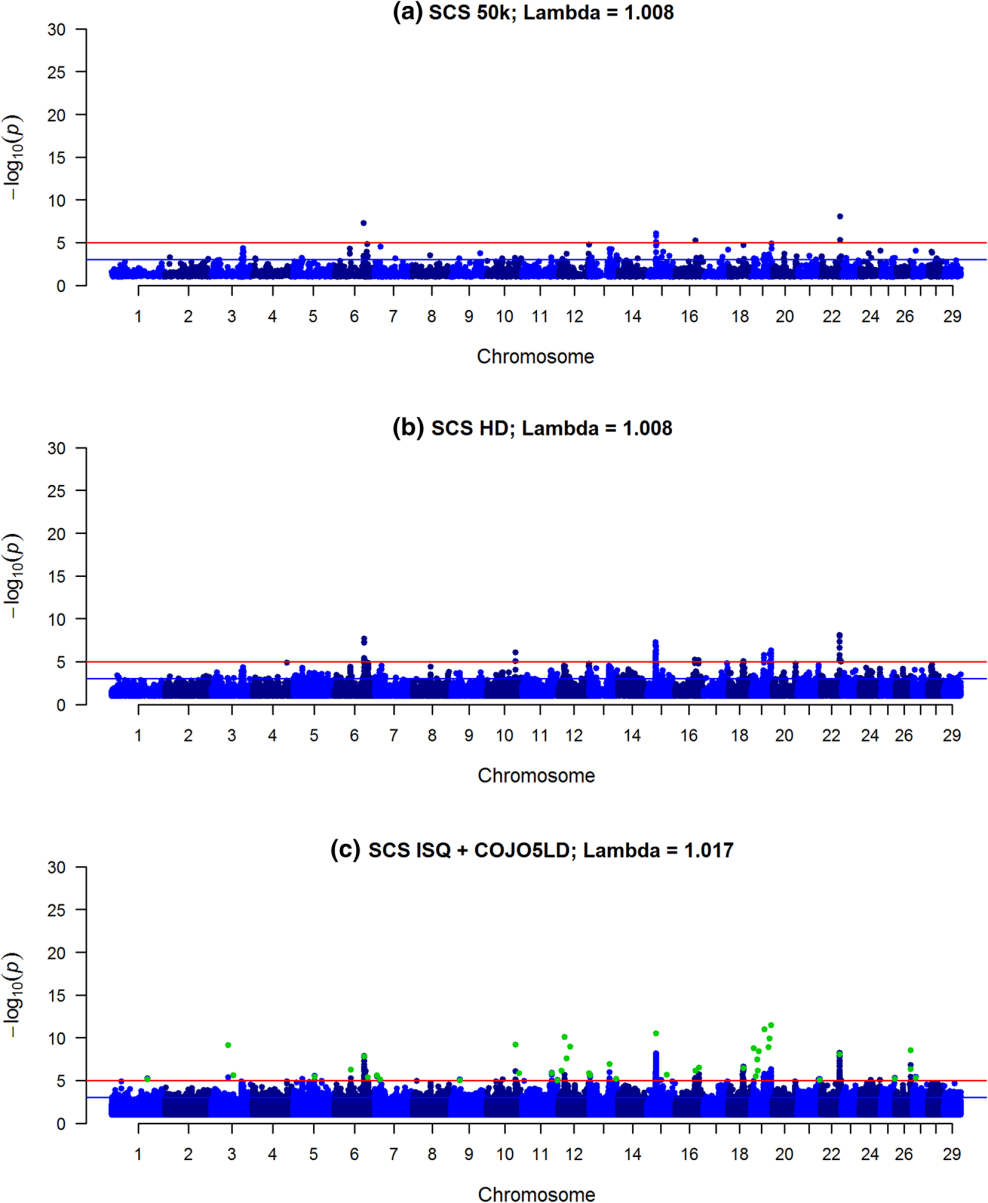
Manhattan plot for somatic cell score (SCS) using Bovine 50k (**a**), BovineHD (**b**) and ISQ data and variants after selection (COJO5LD in *green*) (**c**). Significance of variants effects (−log10(p)) based on the GCTA single variant analyses for somatic cell score (SCS) using Bovine 50k (**a**), BovineHD (**b**), and full sequence data (ISQ) and the variants selected after the COJO5LD analysis (*green*) (**c**)

**Fig. 3:**
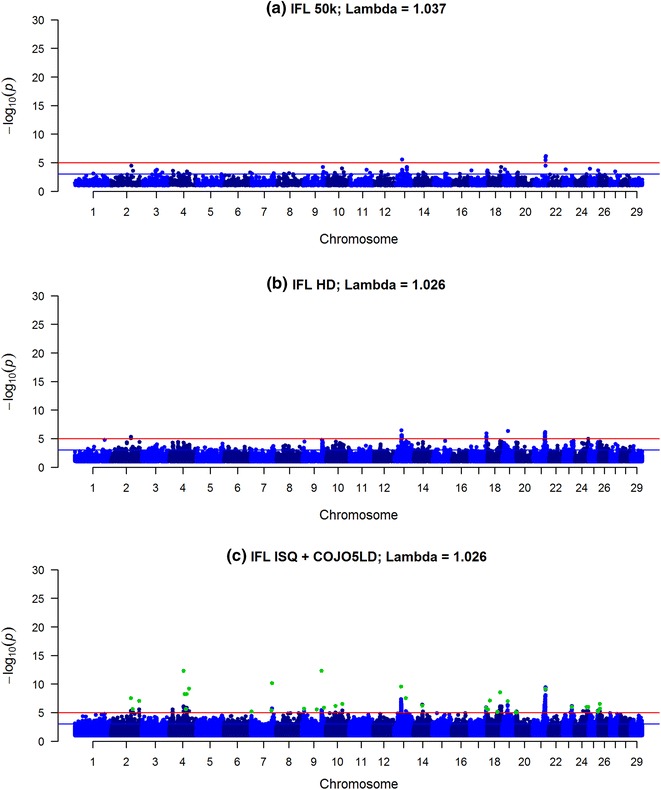
Manhattan plot for interval first last insemination (IFL) using Bovine 50k (**a**), BovineHD (**b**) and ISQ data and variants after selection (COJO5LD in *green*) (**c**). Significance of variants effects (−log10(p)) based on the GCTA single variant analyses for interval between first and last lactation using Bovine 50k (**a**), BovineHD (**b**), and full sequence data (ISQ) and the variants selected after the COJO5LD analysis (*green*) (**c**)

The GWAS results for PY, SCS and IFL are in the Manhattan plots in Figs. 1, 2 and 3 (Q–Q plots are in Additional file 1: Figure S1, Additional file 2: Figure S2, and Additional file 3: Figure S3). Compared to SNP data, when sequence data was used, the peaks became higher and sharper, suggesting a more precise detection of the QTL. For PY, a strong association was found with the *diacylglycerol O*-*acyltransferase 1* (*DGAT1*) gene, and each of the three traits, were associated with several additional variants (−log_10_(p) > 5) when using the imputed sequence data. The total number of variants with a −log_10_(p) value higher than 3 or 5 differed only slightly between traits when using the sequence data (Table 1), and was larger than expected by chance (13,789 and 138 for −log_10_(p) > 3 and −log_10_(p) > 5 respectively). If only the SNPs on the 50k and HD panels were considered, more SNPs with a −log_10_(p) higher than 3 and 5 were found for PY than for SCS and IFL.

In the COJO analysis, variants were selected only when they explained more variance than the other variants already added in the model (starting with the most significant variant). When performing the COJO3 analysis (preselection of variants with a −log_10_(p) > 3), 195 and 264 variants were retained for IFL and SCS, respectively, while for PY only 90 variants were selected (Table 2). However, when performing the COJO5LD analysis (using the more stringent LD criteria of 0.5, i.e. assuming no LD between variants that were more than 100 Mb apart and −log_10_(p) > 5), between 35 and 42 variants were selected and their number for each trait varied little. Many of the remaining variants within the 2-Mb window on either side of the selected variants were in significant LD with the selected variants (as shown in Additional file 4: Figure S4), and up to 3,449,212 variants were excluded from the ${\mathbf{GRM}}{\text{c}}$ (Table 2).

### Variance explained by the selected variants

The ${\mathbf{GRM}}$ of all the variants in the full sequence data resulted in genomic heritabilities (h^2^) of 0.83, 0.87 and 0.72 for PY, SCS and IFL for the validation animals (Table 3), respectively. Compared to these values, the decrease in h^2^ when the HD or 50k SNP panels were used was only marginal (less than 0.04), which indicates that little additional genetic variance is picked up by the sequence data.

**Table 3:** Phenotypic variance (h^2^) explained in 2287 validation animals fitting GRM based on the selected set of variants for protein yield (PY), somatic cell score (SCS) and interval first–last insemination (IFL)

Trait	Selection criteria	ISQ	HD	50k
		Selected set of variants (**GRM**)		
PY	All variants	0.83	0.82	0.81
	−log10(p) > 3	0.53^a^	0.40	0.22
	−log10(p) > 5	0.60^a^	0.43^a^	0.22^a^
	COJO3	0.21		
	COJO5	0.19		
	COJO5LD	0.19		
	COJO#100	0.23		
SCS	All variants	0.87	0.84	0.83
	−log10(p) > 3	0.57^a^	0.45^a^	0.19
	−log10(p) > 5	0.72^a^	0.25^a^	0.03^a^
	COJO3	0.31		
	COJO5	0.31		
	COJO5LD	0.16		
	COJO#100	0.22		
IFL	All variants	0.72	0.70	0.69
	−log10(p) > 3	0.51^a^	0.32	0.14
	−log10(p) > 5	0.50^a^	0.15^a^	0.03
	COJO3	0.25		
	COJO5	0.23		
	COJO5LD	0.13		
	COJO#100	0.17		

ISQ are all imputed sequence variants, and HD and 50k are the SNPs on the common HD and 50k panels. Variants were selected using GWAS results on 3469 discovery animals

^a^Inflated phenotypic variance (see Additional file 5: Table S1)

Selection of variants based on the −log_10_(p) value resulted in fewer variants being used for the ${\mathbf{GRM}}$ and the proportion of variance explained by the ${\mathbf{GRM}}$ was smaller. Exceptions were found for variants selected with a −log_10_(p) > 5 from the full sequence data for PY and SCS; in this case, h^2^ increased with fewer variants compared to when the variants were selected with a −log_10_(p) > 3. However, estimated phenotypic variances were highly inflated for this scenario (see Additional file 5: Table S1): 621 kg^2^, 55 SCS^2^, and 28 d^2^ compared with 308 kg^2^, 19 SCS^2^ and 16 d^2^, for PY, SCS and IFL, respectively. Also, when the lower density SNP panels were used and the variants were strongly selected, and when variants from sequence data were selected with a −log_10_(p) higher than 3, the estimated phenotypic variances were inflated but to a much lesser extent. This inflation of the h^2^ was probably due to the properties of the ${\mathbf{GRM}}$ when it was calculated from a few variants in high LD with each other. Comparing the ${\mathbf{GRM}}$ from HD, 50k, and ISQ −log_10_(p) > 5 variants with the ${\mathbf{GRM}}$ elements from ISQ variants in Additional file 6: Figure S5 and Additional file 7: Table S2, it is clear that the ISQ −log_10_(p) > 5 ${\mathbf{GRM}}$ contained many off-diagonal elements that were equal to the size of the diagonal elements, and therefore, in the analysis, the ${\mathbf{GRM}}$ had to adjust for positive definiteness. When the few variants selected from the COJO analysis were used, h^2^ were not inflated, for example the ${\mathbf{GRM}}$ from the 100 most informative variants in the discovery population resulted in h^2^ that ranged from 0.17 to 0.23 in the validation population. Still, it was clear that even the variants selected from GWAS with (imputed) sequence data could not compete in terms of variance explained with a ${\mathbf{GRM}}$ based on a larger number of variants (e.g. 50k).

Another way of testing the importance of selecting variants was to investigate how much h^2^ was lost when the selected variants were discarded from the ${\mathbf{GRM}}$, i.e. the complementary variants in the ${\mathbf{GRMc}}$. In the scenarios that applied a simple SNP selection, fitting only the ${\mathbf{GRMc}}$ resulted in a drop in h^2^ that was less than 0.01 compared to fitting the full sequence data. With the COJO scenario, the drop in h^2^ compared to fitting the full sequence data was less than 0.04 (results not shown).

To judge the relative importance of the ${\mathbf{GRM}}$ and ${\mathbf{GRMc}}$, both ${\mathbf{GRM}}$ and ${\mathbf{GRMc}}$ were fitted together in a single model (Table 4). For PY, SCS and IFL, in nearly all the scenarios with the p value-based selection, the sum of the two h^2^ was close to the total genetic variance that was explained by the full sequence information (0.83 for PY, 0.87 for SCS, and 0.72 for IFL), except in the analysis with the HD ${\mathbf{GRM}}$ and its ${\mathbf{GRMc}}$, which resulted in highly inflated estimates of h^2^ for PY and IFL. Separately, the HD ${\mathbf{GRM}}$ and the ${\mathbf{GRMc}}$ can both fully explain the genetic variance and among the SNPs that were used to build the ${\mathbf{GRMc}}$, some are probably in strong LD with variants on the HD chip. Consequently, estimation of the genetic variance for these two sets of data with redundant information is probably difficult. For the three traits, the combined effect of ${\mathbf{GRM}}$ and ${\mathbf{GRMc}}$ for −log10(p) > 5 selection from imputed sequence variants (ISQ), i.e. weighting the selected variants differently from the rest of the variants, resulted in a very small improvement of 0.01 for the combined h^2^. In comparison with the p value-based selection, the COJO scenarios resulted in lower h^2^ since many variants were removed from the ${\mathbf{GRMc}}$ on the basis of LD with the selected variants. The 50k panel explained 85% (= 0.70/0.82) of the genetic variance for PY and only 15% was partitioned to the rest of the variants on the sequence, whereas the 50k panel explained 56 and 70% for SCS and IFL. For the COJO analyses, the sum of the two h^2^ was smaller than the total genetic variance that was explained by the full sequence information, but the selected variants from the GWAS accounted for 6 to 18% of the total genetic variance, when they were estimated conditional on the ${\mathbf{GRMc}}$ for the complementary variants.

**Table 4:** Phenotypic variance explained (h^2^) in 2287 validation animals for protein yield (PY), somatic cell score (SCS) and interval first –last insemination (IFL)

Trait	Selection criteria	ISQ	HD	50k	ISQ	HD	50k	ISQ	HD	50k
		Selected set of variants (**GRM**)			Complementary set of variants (**GRM**c)			Sum of **GRM** and **GRM**c		
PY	All variants	0.83	0.98	0.70		0.00	0.12	0.83	0.98	0.82
	−log10(p) > 3	0.19	0.15	0.09	0.61	0.65	0.73	0.80	0.80	0.82
	−log10(p) > 5	0.10	0.04	0.03	0.74	0.79	0.80	0.84	0.83	0.83
	COJO3	0.11			0.70			0.81		
	COJO5	0.10			0.71			0.81		
	COJO5LD	0.08			0.73			0.82		
	COJO#100	0.09			0.72			0.82		
SCS	All variants	0.87	0.87	0.48		0.00	0.38	0.87	0.87	0.86
	−log10(p) > 3	0.22	0.15	0.05	0.60	0.68	0.80	0.82	0.83	0.85
	−log10(p) > 5	0.24	0.03	0.01	0.64	0.84	0.85	0.88	0.87	0.86
	COJO3	0.14			0.68			0.81		
	COJO5	0.14			0.66			0.80		
	COJO5LD	0.05			0.79			0.84		
	COJO#100	0.08			0.76			0.83		
IFL	All variants	0.72	0.85	0.50		0.00	0.21	0.72	0.85	0.70
	−log10(p) > 3	0.20	0.12	0.05	0.51	0.57	0.64	0.70	0.69	0.69
	−log10(p) > 5	0.11	0.03	0.03	0.63	0.69	0.70	0.73	0.72	0.72
	COJO3	0.11			0.57			0.67		
	COJO5	0.12			0.57			0.69		
	COJO5LD	0.07			0.64			0.71		
	COJO#100	0.08			0.64			0.71		

COJO analysis with −log10(p) > 3 (COJO3) or −log10(p) > 5 (COJO5); ISQ are all imputed sequence variants, HD and 50k are the SNPs on the common HD and 50k panels

Variances are estimated fitting ${\mathbf{GRM}}$ and ${\mathbf{GRMc}}$ together in one model where ${\mathbf{GRM}}$ were based on the selected set of variants and ${\mathbf{GRMc}}$ on the complementary variants. Set of variants that were selected using GWAS results on 3469 discovery animals

### Genomic prediction with selected variants

Using ISQ variants or any of the two SNP panels resulted in the same prediction accuracy for PY (0.68), SCS (0.70 to 0.71) and IFL (0.60) (Table 5). Compared to these values, the prediction accuracy for all scenarios decreased when the number of variants in the ${\mathbf{GRM}}$ decreased. However, compared to the simple selection of variants with a −log10(p) > 5, there was a clear advantage in the COJO analyses especially for IFL and to a lesser extent for SCS. For IFL, the prediction accuracy increased from 0.27 for the ${\mathbf{GRM}}$ based on ISQ −log10(p) > 5 to between 0.30 and 0.38 for the COJO scenarios while the number of variants decreased from 853 to between 35 and 264. All variant selection scenarios showed a clear bias (slope <1.0) in the scale of the predictions (Table 5). In general, the bias increased as selection was more stringent and the number of selected variants decreased.

**Table 5:** Prediction accuracy in 2287 validation animals, and the intercept and slope for the regression of phenotype on the breeding value estimated using GRM with different selected sets of variants for protein yield (PY), somatic cell score (SCS) and interval first–last insemination (IFL)

Trait	Selection criteria	ISQ	HD	50k	ISQ	HD	50k	ISQ	HD	50k
		Accuracy			Intercept			Slope		
PY	All variants	0.68	0.68	0.68	−0.6	−0.6	−0.7	0.90	0.90	0.89
	−log10(p) > 3	0.58	0.56	0.42	2.3	3.6	6.9	0.73	0.65	0.57
	−log10(p) > 5	0.39	0.30	0.28	7.2	8.7	9.4	0.54	0.51	0.71
	COJO3	0.40			7.2			0.45		
	COJO5	0.38			7.7			0.41		
	COJO5LD	0.33			8.1			0.51		
	COJO#100	0.34			7.2			0.47		
SCS	All variants	0.70	0.71	0.70	100	100	100	1.02	1.03	1.03
	−log10(p) > 3	0.63	0.55	0.36	100	100	101	0.82	0.79	0.70
	−log10(p) > 5	0.40	0.22	0.11	100	101	101	0.79	0.66	0.57
	COJO3	0.48			100			0.64		
	COJO5	0.48			100			0.60		
	COJO5LD	0.35			100			0.68		
	COJO#100	0.39			100			0.66		
IFL	All variants	0.60	0.60	0.60	99	99	99	0.88	0.87	0.86
	−log10(p) > 3	0.51	0.45	0.31	99	99	99	0.70	0.62	0.52
	−log10(p) > 5	0.27	0.16	0.10	99	98	98	0.47	0.51	0.77
	COJO3	0.38			99			0.45		
	COJO5	0.35			99			0.41		
	COJO5LD	0.30			99			0.52		
	COJO#100	0.32			99			0.45		

ISQ are all imputed sequence variants, HD and 50k are the SNPs on the common HD and 50k panels. Set of variants that were selected using GWAS and subsequently trained in 3469 discovery animals

When both ${\mathbf{GRM}}$ and ${\mathbf{GRMc}}$ are fitted together (Table 6), in the simple variant selection scenarios, all the variants are used but accuracy of genomic prediction may differ between models, because a separate weight is given to the variants in the ${\mathbf{GRM}}$ and ${\mathbf{GRMc}}$. Apart from two of the variant selection scenarios, no increase in accuracy was observed in any scenario when fitting ${\mathbf{GRM}}$ and ${\mathbf{GRMc}}$ compared with fitting the ISQ ${\mathbf{GRM}}$ for all variants. Bias in the prediction was smaller than when each ${\mathbf{GRM}}$ was fitted separately. When variants were selected from HD with −log10(p) > 5 and fitted in a **GRM** separately from the ${\mathbf{GRMc}}$ with the remaining ISQ variants, prediction accuracies for PY and SCS were 0.01 higher than when all ISQ variants were fitted through the ${\mathbf{GRM}}$. This result is similar to that mentioned above for the h^2^, and is in contrast to that of the scenarios fitting −log10(p) > 3 separately, which resulted in lower accuracy and more bias.

**Table 6:** Prediction accuracy in 2287 validation animals, and the intercept and slope for the regression of phenotype on the breeding value estimated using the effects of variants from GRM and GRMc fitted together for different selected sets of variants for protein yield (PY), somatic cell score (SCS) and interval first –last insemination (IFL)

Trait	Selection criteria	ISQ	HD	50k	ISQ	HD	50k	ISQ	HD	50k
		Accuracy			Intercept			Slope		
PY	All variants	0.68	0.68	0.68	−0.6	−0.6	−0.7	0.90	0.90	0.90
	−log10(p) > 3	0.64	0.65	0.67	1.0	1.1	0.4	0.79	0.75	0.83
	−log10(p) > 5	0.67	0.69	0.69	0.2	−0.5	−0.6	0.84	0.90	0.91
	COJO3	0.64			1.4			0.72		
	COJO5	0.64			1.1			0.75		
	COJO5LD	0.67			0.2			0.84		
	COJO#100	0.63			1.2			0.79		
SCS	All variants	0.70	0.71	0.70	100	100	100	1.02	1.03	1.02
	−log10(p) > 3	0.67	0.66	0.66	100	100	100	0.85	0.83	0.87
	−log10(p) > 5	0.67	0.69	0.69	100	100	100	0.93	0.99	1.00
	COJO3	0.63			100			0.76		
	COJO5	0.62			100			0.73		
	COJO5LD	0.65			100			0.88		
	COJO#100	0.62			100			0.83		
IFL	All variants	0.60	0.60	0.60	99	99	99	0.88	0.87	0.87
	−log10(p) > 3	0.55	0.56	0.58	99	99	99	0.73	0.71	0.80
	−log10(p) > 5	0.58	0.61	0.60	99	99	99	0.80	0.88	0.88
	COJO3	0.51			99			0.60		
	COJO5	0.51			99			0.59		
	COJO5LD	0.57			99			0.76		
	COJO#100	0.54			99			0.70		

ISQ are all imputed sequence variants, HD and 50k are the SNPs on the common HD and 50k panels. Set of variants that were selected using GWAS and subsequently trained in 3469 discovery animals

## Discussion

The objective of this study was to identify the usefulness of imputed sequence data, in particular regarding the proportion of variance explained by different sets of variants for three traits PY, SCS and IFL, and for the accuracy of genomic prediction. Full imputed sequence data, lower density SNP panels, and preselected variants from GWAS that used imputed whole-genome sequence were considered. Using the ${\mathbf{GRM}}$ based on full sequence data explained marginally more variation than that based on the common SNP panels. Compared to SNP data, the use of sequence data allowed to identify more variants linked to QTL, and peaks across the genome were sharper, which is in line with other studies using the data of the 1000 Bull Genomes Project [4]. This study clearly showed that the 35 and 42 selected variants from the COJO analysis led to h^2^ that ranged from 0.13 to 0.19 when fitted alone, and from 0.05 to 0.08 when the ${\mathbf{GRM}}$ is competing with a ${\mathbf{GRM}}$ based on the full ISQ information. Thus, such QTL information should be beneficial in genomic prediction. However, no clear benefit for genomic prediction was detected with our data where training and validation populations were both composed of Holstein animals.

### Improving genomic prediction

The fact that sequence data did not improve genomic prediction was previously reported using the same data [8, 28]. In the study of van Binsbergen et al. [8], a Bayesian variable selection method was used with all ISQ variants fitted simultaneously, but the Manhattan plots in that study demonstrated the difficulty of precise QTL detection. QTL were detected, given the prediction accuracy achieved, but the effects were smeared across DNA variants that were in high LD with each other. Calus et al. [28] investigated the split-and-merge approach to alleviate the severe $n \ll p$ problem with sequence data. Neither of these two studies showed an advantage of using sequence data for genomic prediction. Both studies suggested and discussed several explanations for these results and some of these still hold in our study. One explanation concerns the imputed sequence data used, with imputation accuracy being low for some chromosomes. Poor imputation could be due to errors in the genomic map, which reduce accuracy of prediction and detection of causal mutations. Also the training set is relatively small with highly related animals in contrast with the large number of variants available from ISQ. Still, the prior expectation for our study was that, by pre-selecting the ISQ variants some of the limitations (e.g. extreme $p > > n$ and strong LD between many SNPs) would be overcome [11, 29], and more precise QTL detection with ISQ variants would lead to higher accuracy of genomic prediction, especially since ISQ helps to identify more precisely the variants that are associated with the traits [4]. Our results also demonstrated that ISQ helped to identify the QTL, and a limited number of selected variants explained 11 to 14% of the genetic variance, even when fitted with the complementary ${\mathbf{GRMc}}$ at the same time. Hence, there is no doubt that associated regions were identified for all three traits. However, when weighting this prior information in a separate ${\mathbf{GRM}}$, it was difficult to improve the accuracy of genomic prediction compared with the common SNP panels. This confirms the expectation that, within the Holstein population, it is probably difficult to increase the accuracy of prediction, due to the small size of the effective population and the long-range LD in the population, as was previously demonstrated in a simulation study by MacLeod [30]. Hence, using ISQ and selected variants might be especially beneficial for across-breed prediction in small populations [31] or when traits are used with some QTL having large effects, as for fat percentage [32].

### Bias in genomic prediction

Our study showed that the bias in genomic prediction became stronger when variants were strongly preselected. Regressions of DRP on GEBV were expected to be 1, but we found that the stronger the selection of variants was, the stronger the bias was when comparing the predictions in the validation animals. When all ISQ variants or the SNPs on the common panels were fitted in a single ${\mathbf{GRM}}$, the bias in the slope of prediction was limited (slope > 0.86). Also, when ${\mathbf{GRM}}$ and ${\mathbf{GRMc}}$ were fitted together, the bias in the genomic prediction was more controlled. However, when strongly selected variants were used, the genomic predictions became more biased, with slopes even lower than 0.5. Szyda et al. [33] showed that genomic predictions for milk yield were biased when 3 k variants were selected for their effect on milk yield [33]. Brondum et al. [32] reported no extra bias when 1623 selected SNPs were added to the 54 k SNP panel, but when the 1623 SNPs were fitted with their own variance in a model with the 54 k SNPs, the bias increased for some traits.

GEBV could be biased because reported effects of SNPs on a trait tend to be larger in magnitude than the true effects of these SNPs. This phenomenon is well-known, as discussed by Goddard et al. [34]; it is known as the “Beavis effect” [35] and has been described as a form of the “winner’s curse” [36]. The reason underlying the “Beavis effect” is that effects are estimates, and the uncertainty of these estimates is not taken into account. When selecting significant SNPs, we tend to select those for which the uncertainty of the estimate has a positive effect on the variant effect. As pointed out by Goddard et al. [34], when effects of variants are fitted as random effects, this form of the “Beavis effect” is expected to be minimised, since estimates are regressed towards the mean, at least when proper variances are used for each SNP. However, we selected the variants and subsequently trained them for genomic prediction in the same (discovery) population. Thus, the fact that the validation was biased, was a form of the “Beavis effect”: the combined effect of a small set of preselected SNPs was overestimated since the SNPs were selected to have an effect in the same training population.

Another reason for the observed bias is that, rather than the estimates being biased, the bias comes from the overlap between discovery and validation data due to relationships between discovery and validation animals. Overlaps between the validation and discovery data cause bias due to the prediction error covariance between the phenotypes and predictions [37]. Initially, the effect of overlap in the validation and discovery data might be considered as very small here, since validation animals were excluded from both the GWAS and the derivation of the prediction equation, and bulls had highly accurate ${\text{EBV}}$. However, the validation population consisted of animals from subsequent generations of the training animals, e.g. 84% of the validation animals had their sire included in the discovery data [28]. Hence, DNA variants selected from the GWAS were validated within the same families from the discovery population. Also, the phenotypes used are DRP derived from the ${\text{EBV}}$ from national genetic evaluations, in which all records are estimated simultaneously, and therefore the validation animals are strictly speaking not a completely external and independent population. The phenotypic records of the daughters of the young bulls might have also contributed to the breeding values of the sires of the young bulls, and thus result in erroneous correlations between the validation and training or discovery set [38]. The consequence would be that the estimates are not necessarily biased, but the validation was biased due to correlations between the residuals between the training and validation sets.

Which of these underlying reasons is the major cause of the bias observed in our study is not clear. Also, to what extent the bias is controlled by fitting additional variants is not completely clear yet. Given the importance of validation results in practical breeding (i.e. used to assess the scale and reliability of published breeding values), more thought should be given on the validation of genomic breeding values within populations. For example, what are the effects of removing the sires of the validation population on the bias and accuracy when selected SNPs are used, or using separate populations for discovery, training and validation.

### Variant selection

To benefit from the use of sequence data, it needs to be combined with a careful variant selection to pinpoint the QTN [29, 39]. Detection of causal variants based on the data only proved to be difficult due to the large number of variants, and the high LD between variants due to the family relationships in our discovery population. Initially, variant selection was based purely on −log_10_(p) values of the GWAS results. When a high −log_10_(p) threshold was maintained, variants were selected from a few chromosomal regions, but the resulting ${\mathbf{GRM}}$ from these selected variants had poor properties (see Additional file 6: Figure S5 and Additional file 7: Table S2), since the genotypes of too many animals within the population were not sufficiently different for these regions to estimate genetic relationships that differ from 1. Therefore, simply selecting all associated variants was not a good criterion for variant selection. To overcome the issue of selecting variants from the same regions, we used different options of GCTA to do a conditional and joint (COJO) analysis [22]. The first COJO application used only variants pre-selected based on −log_10_(p) value (to reduce the number of variants) but calculated the conditional and joint significance considering LD between all the variants across the whole genome. This variant selection was hindered by collinearity in the data. In spite of excluding from the model variants that were in LD with a r^2^ higher than 0.8, many variants were selected with large effects and −log_10_(p) values, calculated conditional on the joint effects already included in the model. Sometimes the same variants had no effect or effects close to zero −log_10_(p) in the first single variant analyses. Also, when comparing the LD between the variants, the COJO analysis selected few variants that were in moderate to high LD with each other and had opposite effects. Since the estimates were unrealistic, alternative approaches were tested by controlling the LD between variants more stringently. Fewer variants were selected with COJO5LD and only using these in the ${\mathbf{GRM}}$ resulted in lower h^2^ and prediction accuracy, mainly because fewer variants were used. However, combining the COJO5LD together with the ${\mathbf{GRM}}{\text{c}}$ resulted in slightly higher h^2^ and higher prediction accuracy and less bias for all three traits, than any of the other conditional and joint analyses. Thus, when selecting variants, the way that the variants are selected and LD is accounted for is important.

Ideally, Bayesian variable selection methods should be able to separate out the causal variants when all fitted together. However, using the same dataset as in our study, van Binsbergen et al. [8] did not succeed in detecting clear peaks and significance levels for variants in the Manhattan plots when they were fitted all together, and detection of causal variants became even less precise using ISQ in comparison with HD variants [8]. Other studies have combined the biological information available on the functional classes that the variants belong to with the Bayesian variable selection (BAYESRC) [40]. However, in the case of a population with animals as closely related as in our Holstein discovery population, it remains intrinsically difficult to identify the causal variants very precisely.

Brondum et al. [32] performed the GWAS in Nordic cattle for three separate breeds and for three different sets of traits. They selected QTL and three to five variants to tag each QTL and combined 1623 variants with the 50k SNP panel. In contrast to our study, they obtained improved accuracies within the breeds that were used for the GWAS, and the largest improvements in genomic prediction were observed for a French Holstein population that was not used for the GWAS. Also, bias was improved when tested in the independent population of French Holsteins. An extensive study using sequence data, and using across-breed QTL and genomic prediction was performed by van den Berg [41]. Using multibreed information, increases in reliability of up to 10% were found for all the breeds, but they were sensitive to the selection of variants and the model used. In both these studies, LD-pruning was used to select variants [32, 39]. Therefore, the use of sequence information should be accompanied by careful detection and selection of causal variants using concordance analysis or using biological information [29].

## Conclusions

When only the Holstein breed is considered for the discovery of variants and prediction, there is little advantage in using dense sequence data for genomic prediction, although 35 to 42 variants were detected that explained 13 to 19% of the total variation when fitted alone, and 5 to 10% of the variance when they were in competition with other variants. With the within-breed approach, detection of variants and their selection were difficult due to LD. Selection of variants gave more biased genomic predictions. It is unclear if predictions were more biased due to estimating the effects of selected SNPs in the same training population as that from which SNPs were selected, or if the validation was biased due to common family structure or to the use of common data in the national analyses between the training and validation sets.

## Additional files

**Additional file 1: Figure S1.** Q–Q plot for PY using the variants from the Bovine 50k (A), BovineHD (B) and the full imputed sequence data (C).

**Additional file 2: Figure S2.** Q–Q plot for SCS using the variants from the Bovine 50k (A), BovineHD (B) and the full imputed sequence data (C).

**Additional file 3: Figure S3.** Q–Q plot for IFL using the variants from the Bovine 50k (A), BovineHD (B) and the full imputed sequence data (C).

**Additional file 4: Figure S4.** Manhattan plot for three chromosomes and PY using ISQ variants and the variants excluded from **GRMc** based on LD within a 2-Mb window on either side of each selected variant (*green*).

**Additional file 5: Table S1.** Estimated variances, h^2^ and log likelihood for PY, SCS and IFL using models with different single **GRM**, or **GRM** + **GRMc** combined in one model. Description: **GRM** were based on SNPs selected from GWAS. Standard errors are below the estimates.

**Additional file 6: Figure S5.** Plot of the elements of a **GRM** based on the 50k SNPs, −log10(p) > 5 and COJO5LD versus the same elements using ISQ.

**Additional file 7: Table S2.** Summary statistics for the diagonal and off diagonal elements of the different **GRM**.
