# Supplementary figures and images for: Genomic prediction using preselected DNA variants from a GWAS with whole-genome sequence data in Holstein–Friesian cattle
Source: Genet Sel Evol. 2016 Dec 1;48:95. doi: 10.1186/s12711-016-0274-1 (PMC5134274; doi:10.1186/s12711-016-0274-1)

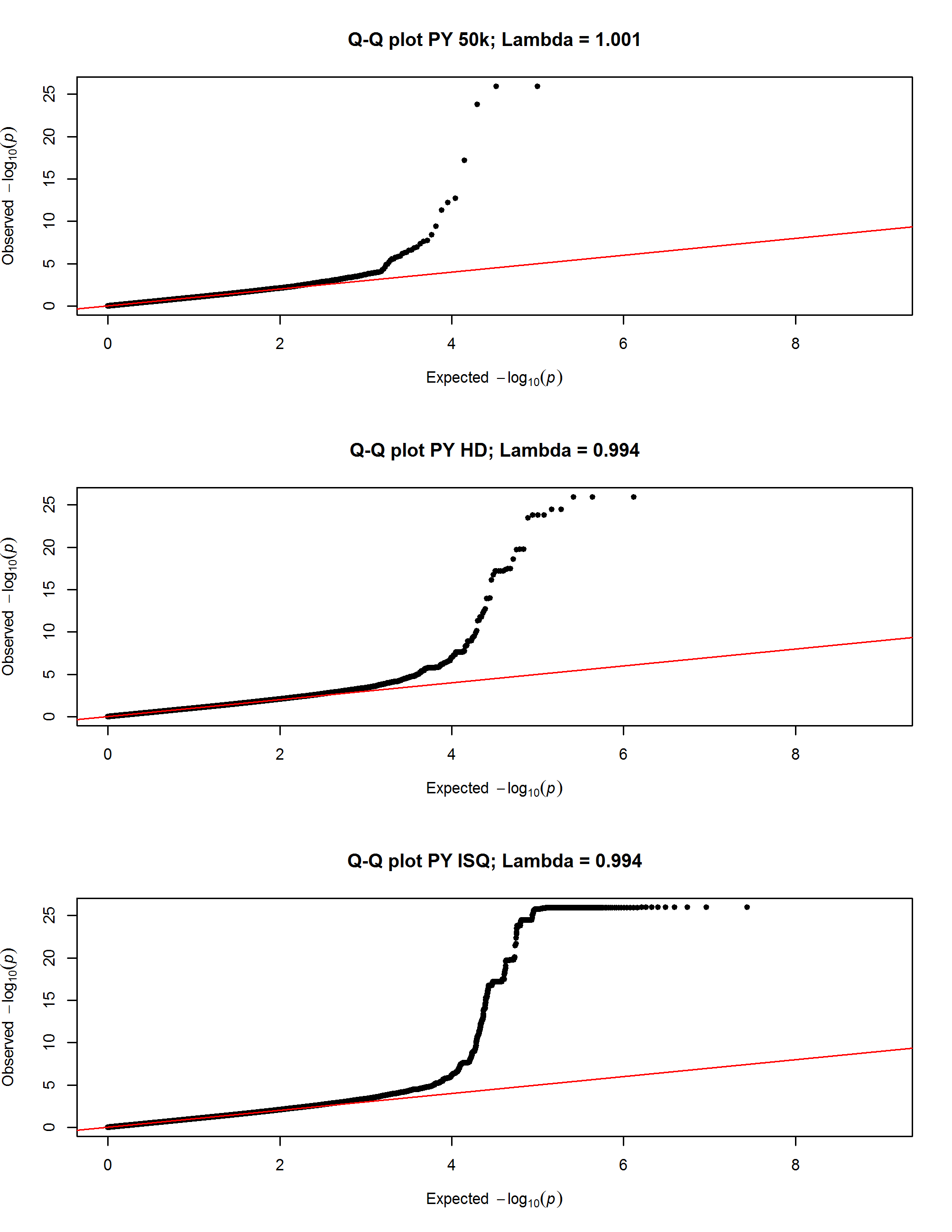

Supplement: Supplementary file 1 — Additional file 1: Figure S1. Q–Q plot for PY using the variants from the Bovine 50k (A), BovineHD (B) and the full imputed sequence data (C). [file 12711_2016_274_MOESM1_ESM.png]

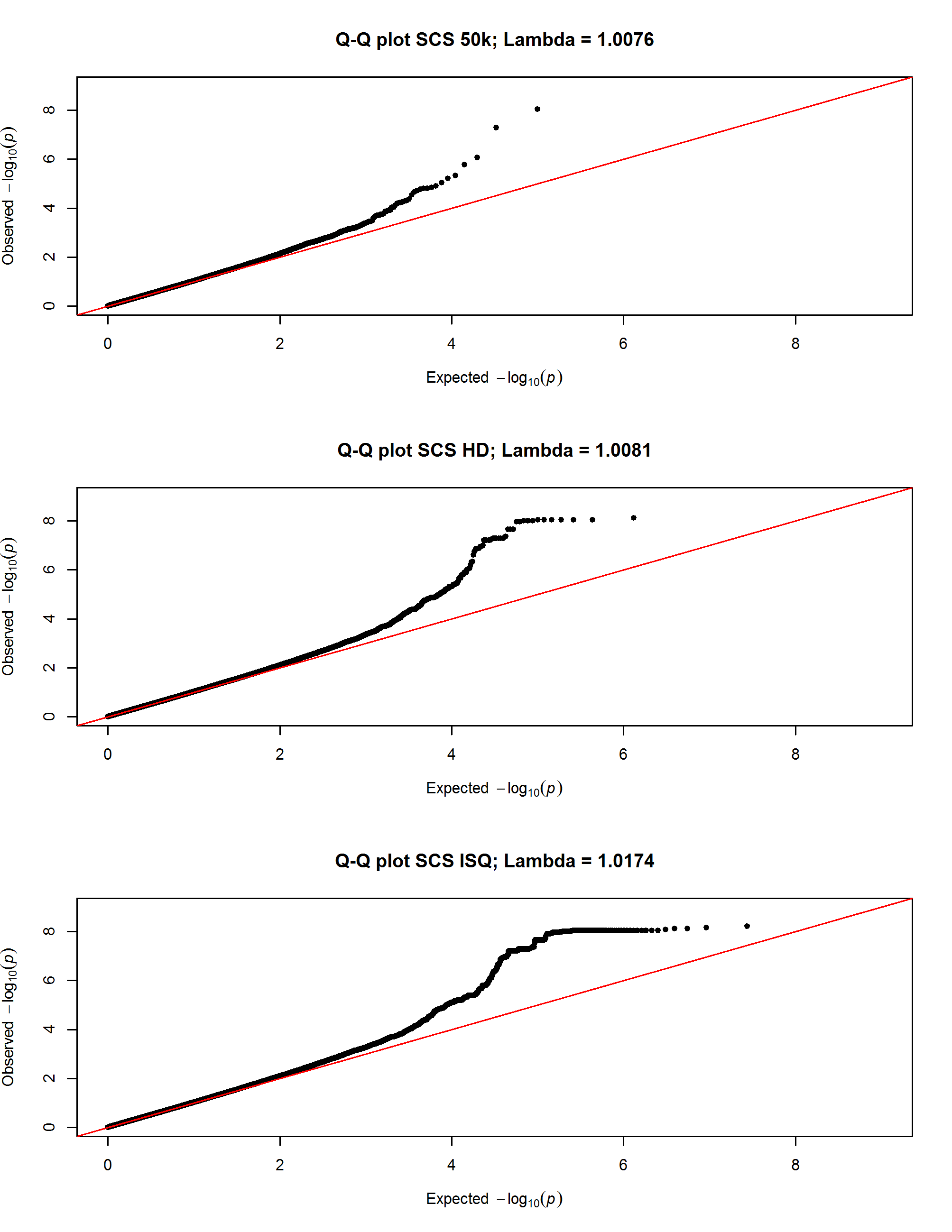

Supplement: Supplementary file 2 — Additional file 2: Figure S2. Q–Q plot for SCS using the variants from the Bovine 50k (A), BovineHD (B) and the full imputed sequence data (C). [file 12711_2016_274_MOESM2_ESM.png]

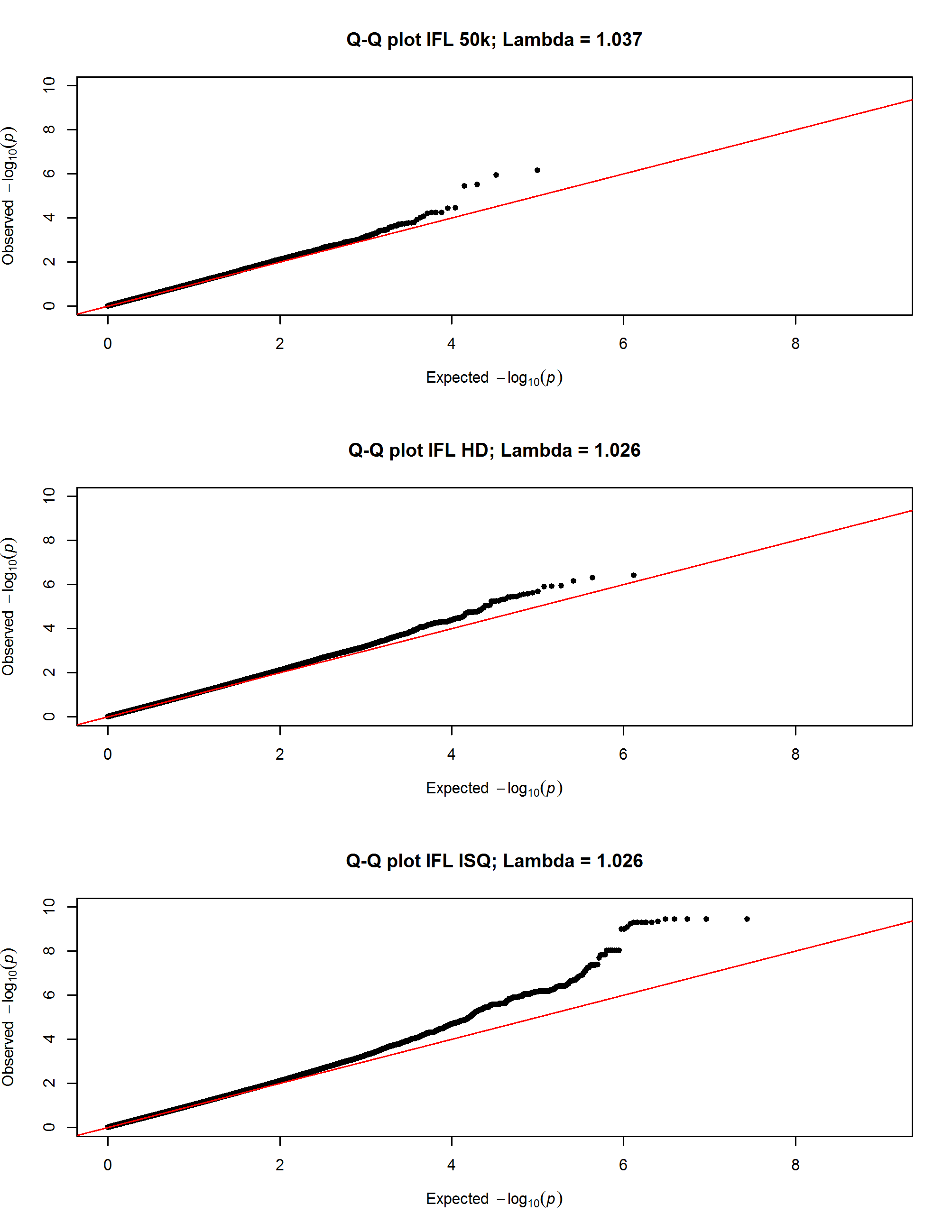

Supplement: Supplementary file 3 — Additional file 3: Figure S3. Q–Q plot for IFL using the variants from the Bovine 50k (A), BovineHD (B) and the full imputed sequence data (C). [file 12711_2016_274_MOESM3_ESM.png]

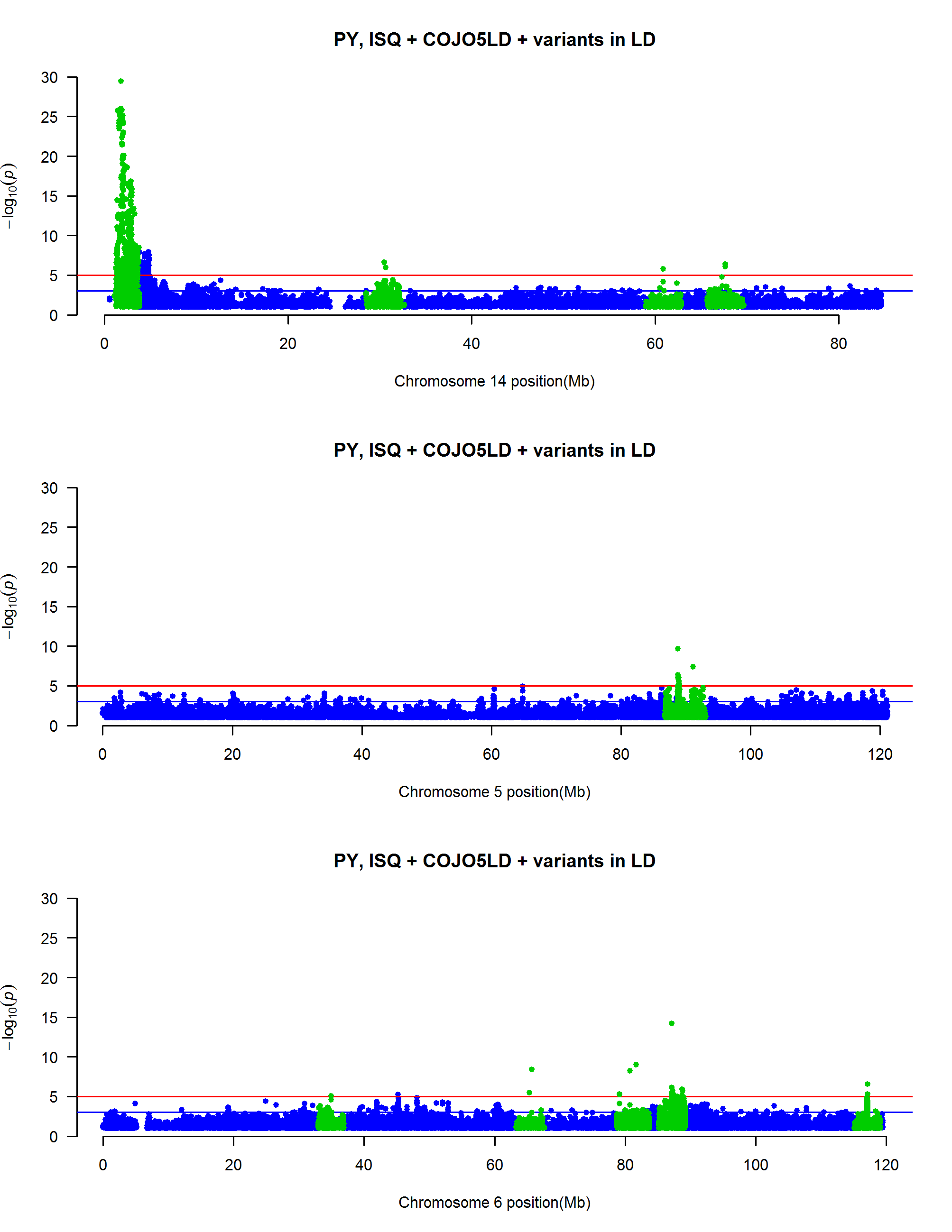

Supplement: Supplementary file 4 — Additional file 4: Figure S4. Manhattan plot for three chromosomes and PY using ISQ variants and the variants excluded from GRMc based on LD within a 2-Mb window on either side of each selected variant (green). [file 12711_2016_274_MOESM4_ESM.png]

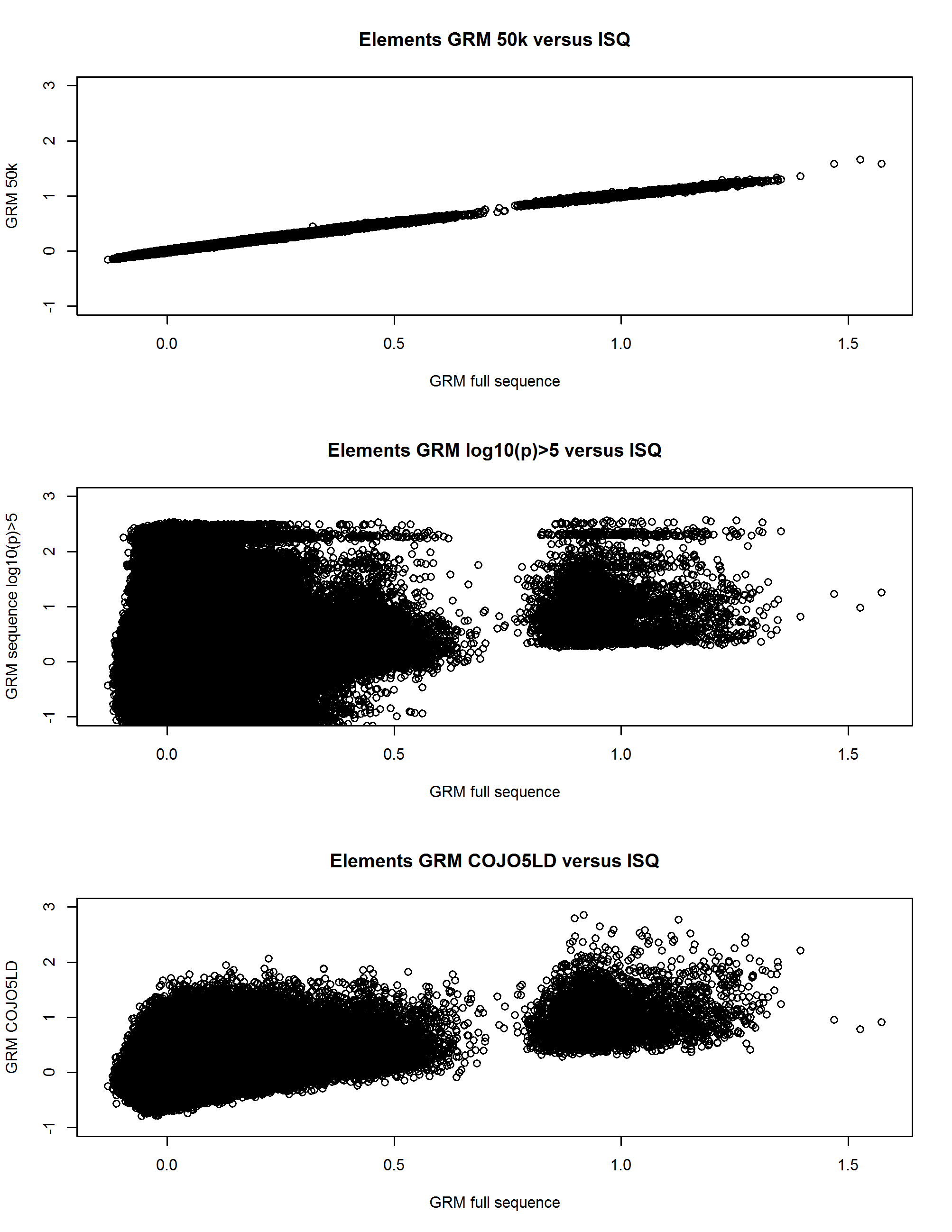

Supplement: Supplementary file 6 — Additional file 6: Figure S5. Plot of the elements of a GRM based on the 50k SNPs, −log10(p) > 5 and COJO5LD versus the same elements using ISQ. [file 12711_2016_274_MOESM6_ESM.png]
