# Supplementary material for: Genomic prediction using preselected DNA variants from a GWAS with whole-genome sequence data in Holstein–Friesian cattle
Source: Genet Sel Evol. 2016 Dec 1;48:95. doi: 10.1186/s12711-016-0274-1 (PMC5134274; doi:10.1186/s12711-016-0274-1)
Supplement: Supplementary file 5 — Additional file 5: Table S1. Estimated variances, h2 and log likelihood for PY, SCS and IFL using models with different single GRM, or GRM + GRMc combined in one model. Description: GRM were based on SNPs selected from GWAS. Standard errors are below the estimates. [file 12711_2016_274_MOESM5_ESM.docx]

Additional file 1:

Table 7 Estimated variances, h^2^ and log likelihood for PY, SCS and IFL using models with different single GRM, or GRM+GRMc combined in one model. GRM were based on selected SNP from GWAS. Standard errors are below the estimates.

**PY:**

|  | V_GRM_ | V_GRMc_ | Ve | Vp | V_GRM_ /Vp | V_GRMc_ /Vp | (V_GRM_ + V_GRMc)_  /Vp | logL  base = -**7245.96** |
| --- | --- | --- | --- | --- | --- | --- | --- | --- |
| 50k | 249.8 |  | 57.4 | 307.2 | 0.81 |  |  | -6751.8 |
|  | 16.5 |  | 6.6 | 12.5 | 0.03 |  |  |  |
| 50k -log10(p)>3 | 66.8 |  | 233.6 | 300.4 | 0.22 |  |  | -6982.5 |
|  | 12.2 |  | 7.3 | 13.9 | 0.03 |  |  |  |
| 50k -log10(p)>5 | 79.0 |  | 283.5 | 362.4 | 0.22 |  |  | -7134.1 |
|  | 31.1 |  | 8.7 | 32.1 | 0.07 |  |  |  |
| HD | 248.9 |  | 56.4 | 305.3 | 0.82 |  |  | -6752.6 |
|  | 16.5 |  | 6.7 | 12.4 | 0.03 |  |  |  |
| HD -log10(p)>3 | 121.6 |  | 182.5 | 304.1 | 0.40 |  |  | -6846.4 |
|  | 14.7 |  | 6.1 | 14.9 | 0.03 |  |  |  |
| HD -log10(p)>5 | 206.0 |  | 271.0 | 477.0 | 0.43 |  |  | -7109.5 |
|  | 63.4 |  | 8.4 | 63.3 | 0.08 |  |  |  |
| ISQ | 256.7 |  | 52.1 | 308.8 | 0.83 |  |  | -6765.0 |
|  | 17.2 |  | 7.2 | 12.6 | 0.03 |  |  |  |
| ISQ -log10(p)>3 | 173.9 |  | 156.0 | 329.9 | 0.53 |  |  | -6797.2 |
|  | 19.3 |  | 6.0 | 18.0 | 0.03 |  |  |  |
| ISQ -log10(p)>5 | 375.3 |  | 246.1 | 621.4 | 0.60 |  |  | -7049.1 |
|  | 79.1 |  | 7.8 | 78.5 | 0.05 |  |  |  |
| COJO3 | 61.9 |  | 238.6 | 300.5 | 0.21 |  |  | -7008.6 |
|  | 10.8 |  | 7.4 | 13.0 | 0.03 |  |  |  |
| COJO5 | 55.5 |  | 243.7 | 299.2 | 0.19 |  |  | -7014.5 |
|  | 11.1 |  | 7.6 | 13.4 | 0.03 |  |  |  |
| COJO5LD | 60.7 |  | 263.7 | 324.4 | 0.19 |  |  | -7077.7 |
|  | 15.9 |  | 8.1 | 17.7 | 0.04 |  |  |  |
| COJO#100 | 73.2 |  | 242.5 | 315.7 | 0.23 |  |  | -7034.0 |
|  | 12.6 |  | 7.6 | 14.5 | 0.03 |  |  |  |
| 50k | 214.3 | 38.3 | 55.2 | 307.7 | 0.70 | 0.12 | 0.82 | -6751.4 |
|  | 43.2 | 44.1 | 7.1 | 12.6 | 0.14 | 0.14 | 0.03 |  |
| 50k -log10(p)>3 | 25.5 | 217.0 | 53.5 | 296.0 | 0.09 | 0.73 | 0.82 | -6695.5 |
|  | 6.2 | 15.6 | 6.8 | 12.5 | 0.02 | 0.03 | 0.03 |  |
| 50k -log10(p)>5 | 8.6 | 239.4 | 51.0 | 299.1 | 0.03 | 0.80 | 0.83 | -6716.3 |
|  | 4.4 | 16.2 | 6.9 | 12.5 | 0.01 | 0.03 | 0.03 |  |
| HD | 306.3 | 0.0 | 6.9 | 313.2 | 0.98 | 0.00 | 0.98 | -6811.4 |
|  | 50.3 | 50.9 | 4.3 | 12.3 | 0.16 | 0.16 | 0.01 |  |
| HD -log10(p)>3 | 43.7 | 190.1 | 57.2 | 291.0 | 0.15 | 0.65 | 0.80 | -6678.7 |
|  | 8.3 | 15.2 | 6.7 | 12.3 | 0.03 | 0.04 | 0.03 |  |
| HD -log10(p)>5 | 11.6 | 238.7 | 50.9 | 301.2 | 0.04 | 0.79 | 0.83 | -6716.1 |
|  | 5.9 | 16.2 | 6.8 | 13.0 | 0.02 | 0.03 | 0.03 |  |
| ISQ -log10(p)>3 | 54.0 | 179.1 | 58.2 | 291.3 | 0.19 | 0.61 | 0.80 | -6675.3 |
|  | 10.1 | 15.2 | 6.8 | 12.5 | 0.03 | 0.04 | 0.03 |  |
| ISQ -log10(p)>5 | 30.9 | 231.0 | 51.9 | 313.9 | 0.10 | 0.74 | 0.83 | -6711.1 |
|  | 13.7 | 16.0 | 6.8 | 16.9 | 0.04 | 0.04 | 0.03 |  |
| COJO3 | 33.7 | 210.8 | 56.8 | 301.3 | 0.11 | 0.70 | 0.81 | -6709.0 |
|  | 6.9 | 15.4 | 6.7 | 12.8 | 0.02 | 0.03 | 0.03 |  |
| COJO5 | 29.0 | 212.4 | 58.1 | 299.4 | 0.10 | 0.71 | 0.81 | -6711.8 |
|  | 6.7 | 15.5 | 6.9 | 12.8 | 0.02 | 0.03 | 0.03 |  |
| COJO5LD | 25.6 | 225.2 | 55.6 | 306.4 | 0.08 | 0.73 | 0.82 | -6723.3 |
|  | 7.4 | 15.9 | 6.9 | 13.5 | 0.02 | 0.03 | 0.03 |  |
| COJO#100 | 28.5 | 222.4 | 56.4 | 307.2 | 0.09 | 0.72 | 0.82 | -6739.4 |
|  | 6.2 | 16.1 | 7.0 | 12.8 | 0.02 | 0.03 | 0.03 |  |
| 50k+COJO5LD | 217.6 | 22.9 | 61.6 | 302.2 | 0.72 | 0.08 | 0.80 | -6715.5 |
|  | 15.3 | 6.9 | 6.4 | 13.2 | 0.03 | 0.02 | 0.03 |  |
| HD+COJO5LD | 220.0 | 23.7 | 58.5 | 302.2 | 0.73 | 0.08 | 0.81 | -6710.6 |
|  | 15.3 | 7.1 | 6.4 | 13.2 | 0.03 | 0.02 | 0.03 |  |

**SCS:**

|  | V_GRM_ | V_GRMc_ | Ve | Vp | V_GRM_ /Vp | V_GRMc_ /Vp | (V_GRM_ + V_GRMc)_  /Vp | logL  base = 4302.85 |
| --- | --- | --- | --- | --- | --- | --- | --- | --- |
| 50k | 16.0 |  | 3.3 | 19.3 | 0.83 |  |  | -3773.4 |
|  | 1.0 |  | 0.4 | 0.8 | 0.02 |  |  |  |
| 50k -log10(p)>3 | 3.8 |  | 16.2 | 19.9 | 0.19 |  |  | -4123.6 |
|  | 0.7 |  | 0.5 | 0.8 | 0.03 |  |  |  |
| 50k -log10(p)>5 | 0.6 |  | 20.0 | 20.6 | 0.03 |  |  | -4278.9 |
|  | 0.4 |  | 0.6 | 0.7 | 0.02 |  |  |  |
| HD | 16.2 |  | 3.0 | 19.3 | 0.84 |  |  | -3766.5 |
|  | 1.0 |  | 0.4 | 0.8 | 0.02 |  |  |  |
| HD -log10(p)>3 | 9.5 |  | 11.6 | 21.2 | 0.45 |  |  | -3926.0 |
|  | 1.1 |  | 0.4 | 1.1 | 0.03 |  |  |  |
| HD -log10(p)>5 | 6.1 |  | 18.8 | 24.9 | 0.25 |  |  | -4239.6 |
|  | 2.2 |  | 0.6 | 2.3 | 0.07 |  |  |  |
| ISQ | 17.1 |  | 2.6 | 19.7 | 0.87 |  |  | -3776.3 |
|  | 1.1 |  | 0.4 | 0.8 | 0.03 |  |  |  |
| ISQ -log10(p)>3 | 12.7 |  | 9.7 | 22.4 | 0.57 |  |  | -3853.6 |
|  | 1.3 |  | 0.4 | 1.3 | 0.03 |  |  |  |
| ISQ -log10(p)>5 | 39.8 |  | 15.4 | 55.3 | 0.72 |  |  | -4102.2 |
|  | 7.7 |  | 0.5 | 7.7 | 0.04 |  |  |  |
| COJO3 | 6.2 |  | 13.7 | 20.0 | 0.31 |  |  | -4028.4 |
|  | 0.8 |  | 0.4 | 0.9 | 0.03 |  |  |  |
| COJO5 | 6.0 |  | 13.4 | 19.4 | 0.31 |  |  | -4006.4 |
|  | 0.8 |  | 0.4 | 0.8 | 0.03 |  |  |  |
| COJO5LD | 3.3 |  | 17.3 | 20.6 | 0.16 |  |  | -4162.9 |
|  | 0.8 |  | 0.5 | 1.0 | 0.03 |  |  |  |
| COJO#100 | 4.4 |  | 15.7 | 20.1 | 0.22 |  |  | -4104.7 |
|  | 0.8 |  | 0.5 | 0.9 | 0.03 |  |  |  |
| 50k | 9.3 | 7.4 | 2.8 | 19.5 | 0.48 | 0.38 | 0.86 | -3769.6 |
|  | 2.6 | 2.8 | 0.4 | 0.8 | 0.13 | 0.14 | 0.03 |  |
| 50k -log10(p)>3 | 0.9 | 15.3 | 2.9 | 19.1 | 0.05 | 0.80 | 0.85 | -3757.7 |
|  | 0.3 | 1.0 | 0.4 | 0.8 | 0.01 | 0.03 | 0.03 |  |
| 50k -log10(p)>5 | 0.2 | 16.7 | 2.6 | 19.6 | 0.01 | 0.85 | 0.87 | -3769.8 |
|  | 0.2 | 1.1 | 0.4 | 0.8 | 0.01 | 0.03 | 0.03 |  |
| HD | 16.7 | 0.0 | 2.6 | 19.3 | 0.87 | 0.00 | 0.87 | -3767.3 |
|  | 3.6 | 3.7 | 0.4 | 0.8 | 0.19 | 0.19 | 0.02 |  |
| HD -log10(p)>3 | 2.9 | 12.9 | 3.3 | 19.0 | 0.15 | 0.68 | 0.83 | -3733.6 |
|  | 0.6 | 1.0 | 0.4 | 0.8 | 0.03 | 0.04 | 0.03 |  |
| HD -log10(p)>5 | 0.5 | 16.4 | 2.7 | 19.6 | 0.03 | 0.84 | 0.86 | -3766.5 |
|  | 0.3 | 1.1 | 0.4 | 0.8 | 0.02 | 0.03 | 0.03 |  |
| ISQ -log10(p)>3 | 4.1 | 11.5 | 3.4 | 19.1 | 0.22 | 0.60 | 0.82 | -3719.8 |
|  | 0.7 | 1.0 | 0.4 | 0.8 | 0.03 | 0.04 | 0.03 |  |
| ISQ -log10(p)>5 | 5.5 | 15.0 | 2.9 | 23.4 | 0.24 | 0.64 | 0.88 | -3752.8 |
|  | 1.9 | 1.0 | 0.4 | 1.9 | 0.06 | 0.06 | 0.02 |  |
| COJO3 | 2.7 | 13.0 | 3.6 | 19.3 | 0.14 | 0.68 | 0.81 | -3763.9 |
|  | 0.5 | 1.0 | 0.4 | 0.8 | 0.02 | 0.03 | 0.03 |  |
| COJO5 | 2.7 | 12.6 | 3.8 | 19.0 | 0.14 | 0.66 | 0.80 | -3764.1 |
|  | 0.4 | 1.0 | 0.4 | 0.8 | 0.02 | 0.03 | 0.03 |  |
| COJO5LD | 1.0 | 15.4 | 3.0 | 19.4 | 0.05 | 0.79 | 0.84 | -3763.1 |
|  | 0.3 | 1.1 | 0.4 | 0.8 | 0.02 | 0.03 | 0.03 |  |
| COJO#100 | 1.5 | 14.6 | 3.2 | 19.3 | 0.08 | 0.76 | 0.83 | -3763.9 |
|  | 0.3 | 1.0 | 0.4 | 0.8 | 0.02 | 0.03 | 0.03 |  |
| 50k+COJO5LD | 14.5 | 0.9 | 3.5 | 19.0 | 0.76 | 0.05 | 0.81 | -3756.6 |
|  | 1.0 | 0.3 | 0.4 | 0.8 | 0.03 | 0.02 | 0.03 |  |
| HD+COJO5LD | 14.8 | 0.9 | 3.3 | 19.0 | 0.78 | 0.05 | 0.83 | -3751.4 |
|  | 1.0 | 0.3 | 0.4 | 0.8 | 0.03 | 0.01 | 0.03 |  |

**IFL:**

|  | V_GRM_ | V_GRMc_ | Ve | Vp | V_GRM_ /Vp | V_GRMc_ /Vp | (V_GRM_ + V_GRMc)_  /Vp | logL  base = 4055.60 |
| --- | --- | --- | --- | --- | --- | --- | --- | --- |
| 50k | 11.0 |  | 5.0 | 16.1 | 0.69 |  |  | -3721.0 |
|  | 0.9 |  | 0.4 | 0.6 | 0.03 |  |  |  |
| 50k -log10(p)>3 | 2.2 |  | 13.7 | 15.9 | 0.14 |  |  | -3919.3 |
|  | 0.5 |  | 0.4 | 0.6 | 0.03 |  |  |  |
| 50k -log10(p)>5 | 0.5 |  | 16.1 | 16.6 | 0.03 |  |  | -4046.4 |
|  | 0.6 |  | 0.5 | 0.7 | 0.03 |  |  |  |
| HD | 11.2 |  | 4.8 | 16.0 | 0.70 |  |  | -3716.5 |
|  | 0.9 |  | 0.4 | 0.6 | 0.03 |  |  |  |
| HD -log10(p)>3 | 5.3 |  | 11.2 | 16.5 | 0.32 |  |  | -3827.0 |
|  | 0.7 |  | 0.4 | 0.8 | 0.03 |  |  |  |
| HD -log10(p)>5 | 2.7 |  | 15.6 | 18.3 | 0.15 |  |  | -4024.9 |
|  | 1.4 |  | 0.5 | 1.4 | 0.06 |  |  |  |
| ISQ | 11.8 |  | 4.6 | 16.3 | 0.72 |  |  | -3726.1 |
|  | 0.9 |  | 0.4 | 0.7 | 0.03 |  |  |  |
| ISQ -log10(p)>3 | 9.5 |  | 9.3 | 18.9 | 0.51 |  |  | -3772.1 |
|  | 1.1 |  | 0.4 | 1.0 | 0.03 |  |  |  |
| ISQ -log10(p)>5 | 14.1 |  | 14.2 | 28.3 | 0.50 |  |  | -3975.1 |
|  | 3.2 |  | 0.4 | 3.2 | 0.06 |  |  |  |
| COJO3 | 3.9 |  | 11.8 | 15.7 | 0.25 |  |  | -3855.0 |
|  | 0.5 |  | 0.4 | 0.6 | 0.03 |  |  |  |
| COJO5 | 3.6 |  | 12.3 | 16.0 | 0.23 |  |  | -3884.0 |
|  | 0.5 |  | 0.4 | 0.6 | 0.03 |  |  |  |
| COJO5LD | 2.2 |  | 14.4 | 16.6 | 0.13 |  |  | -3959.0 |
|  | 0.6 |  | 0.4 | 0.7 | 0.03 |  |  |  |
| COJO#100 | 2.8 |  | 13.7 | 16.5 | 0.17 |  |  | -3945.8 |
|  | 0.5 |  | 0.4 | 0.7 | 0.03 |  |  |  |
| 50k | 8.0 | 3.3 | 4.8 | 16.1 | 0.50 | 0.21 | 0.70 | -3720.1 |
|  | 2.4 | 2.5 | 0.4 | 0.6 | 0.15 | 0.15 | 0.03 |  |
| 50k -log10(p)>3 | 0.8 | 10.2 | 4.9 | 15.9 | 0.05 | 0.64 | 0.69 | -3704.3 |
|  | 0.3 | 0.9 | 0.4 | 0.6 | 0.02 | 0.04 | 0.03 |  |
| 50k -log10(p)>5 | 0.4 | 11.5 | 4.6 | 16.5 | 0.03 | 0.70 | 0.72 | -3717.1 |
|  | 0.4 | 0.9 | 0.4 | 0.8 | 0.03 | 0.04 | 0.03 |  |
| HD | 13.6 | 0.0 | 2.4 | 16.0 | 0.85 | 0.00 | 0.85 | -3746.8 |
|  | 3.0 | 3.1 | 0.3 | 0.7 | 0.19 | 0.19 | 0.02 |  |
| HD -log10(p)>3 | 1.9 | 9.1 | 4.9 | 15.9 | 0.12 | 0.57 | 0.69 | -3688.1 |
|  | 0.4 | 0.8 | 0.4 | 0.7 | 0.03 | 0.04 | 0.03 |  |
| HD -log10(p)>5 | 0.4 | 11.3 | 4.6 | 16.3 | 0.03 | 0.69 | 0.72 | -3712.7 |
|  | 0.3 | 0.9 | 0.4 | 0.7 | 0.02 | 0.04 | 0.03 |  |
| ISQ -log10(p)>3 | 3.2 | 8.3 | 4.8 | 16.4 | 0.20 | 0.51 | 0.70 | -3679.1 |
|  | 0.6 | 0.8 | 0.4 | 0.7 | 0.03 | 0.04 | 0.03 |  |
| ISQ -log10(p)>5 | 1.8 | 10.8 | 4.6 | 17.2 | 0.11 | 0.63 | 0.73 | -3699.0 |
|  | 0.8 | 0.9 | 0.4 | 1.0 | 0.04 | 0.04 | 0.03 |  |
| COJO3 | 1.7 | 9.0 | 5.2 | 15.9 | 0.11 | 0.57 | 0.67 | -3709.4 |
|  | 0.3 | 0.8 | 0.4 | 0.6 | 0.02 | 0.04 | 0.03 |  |
| COJO5 | 1.9 | 9.2 | 5.0 | 16.1 | 0.12 | 0.57 | 0.69 | -3704.7 |
|  | 0.4 | 0.8 | 0.4 | 0.7 | 0.02 | 0.04 | 0.03 |  |
| COJO5LD | 1.1 | 10.5 | 4.7 | 16.3 | 0.07 | 0.64 | 0.71 | -3698.5 |
|  | 0.3 | 0.9 | 0.4 | 0.7 | 0.02 | 0.04 | 0.03 |  |
| COJO#100 | 1.2 | 10.4 | 4.7 | 16.4 | 0.08 | 0.64 | 0.71 | -3711.4 |
|  | 0.3 | 0.9 | 0.4 | 0.7 | 0.02 | 0.04 | 0.03 |  |
| 50k+COJO5LD | 10.0 | 1.0 | 5.0 | 16.1 | 0.62 | 0.06 | 0.69 | -3692.9 |
|  | 0.8 | 0.3 | 0.4 | 0.7 | 0.03 | 0.02 | 0.03 |  |
| HD+COJO5LD | 10.1 | 1.0 | 4.9 | 16.0 | 0.63 | 0.06 | 0.70 | -3689.0 |
|  | 0.8 | 0.3 | 0.4 | 0.7 | 0.04 | 0.02 | 0.03 |  |
