# Supplementary material for: Genomic prediction using preselected DNA variants from a GWAS with whole-genome sequence data in Holstein–Friesian cattle
Source: Genet Sel Evol. 2016 Dec 1;48:95. doi: 10.1186/s12711-016-0274-1 (PMC5134274; doi:10.1186/s12711-016-0274-1)
Supplement: Supplementary file 7 — Additional file 7: Table S2. Summary statistics for the diagonal and off diagonal elements of the different GRM. [file 12711_2016_274_MOESM7_ESM.docx]

Additional file 2: Properties of the GRM

Table 8: Summary statistics for the diagonal and off diagonal elements of the different GRM.

| GRM |  | Min. | 1st Qu. | Median | Mean | 3rd Qu. | Max. |
| --- | --- | --- | --- | --- | --- | --- | --- |
| ISQ | Diagonals | 0.699 | 0.902 | 0.941 | 0.962 | 0.998 | 1.574 |
| HD | Diagonals | 0.756 | 0.933 | 0.966 | 0.983 | 1.012 | 1.648 |
| 50k | Diagonals | 0.749 | 0.933 | 0.966 | 0.983 | 1.014 | 1.661 |
| ISQ -log(p)>5 | Diagonals | 0.258 | 0.552 | 0.888 | 0.944 | 1.164 | 2.578 |
| COJO5LD | Diagonals | 0.283 | 0.701 | 0.892 | 0.959 | 1.145 | 2.855 |
| ISQ | Off-diagonals | -0.131 | -0.023 | -0.007 | 0.000 | 0.013 | 1.074 |
| HD | Off-diagonals | -0.155 | -0.025 | -0.007 | 0.000 | 0.015 | 1.087 |
| 50k | Off-diagonals | -0.156 | -0.025 | -0.007 | 0.000 | 0.016 | 1.076 |
| ISQ -log(p)>5 | Off-diagonals | -1.566 | -0.279 | 0.000 | 0.000 | 0.287 | 2.534 |
| COJO5LD | Off-diagonals | -0.790 | -0.117 | -0.013 | 0.000 | 0.100 | 2.066 |
